# Fatty acid binding protein 4 in circulating leucocytes reflects atherosclerotic lesion progression in *Apoe*^−/−^ mice

**DOI:** 10.1111/jcmm.12011

**Published:** 2013-02-07

**Authors:** Hanna E Agardh, Karl Gertow, Dolores M Salvado, Andreas Hermansson, Gijs H Puijvelde, Göran K Hansson, Gabrielle Paulsso n-Berne, Anders Gabrielsen

**Affiliations:** a Experimental Cardiovascular Research, Department of Medicine, Center for Molecular Medicine, Karolinska InstitutetStockholm, Sweden; b Division of Chemistry 2, Department of Medical Biochemistry and Biophysics, Karolinska InstitutetStockholm, Sweden; c Division of Biopharmaceutics, Leiden/Amsterdam Center for Drug Research, Gorlaeus LaboratoriesLeiden, The Netherlands

**Keywords:** atherosclerosis, biomarkers, leucocytes

## Abstract

Discovery of novel biomarkers for atherosclerosis is important to aid in early diagnosis of pre-symptomatic patients at high risk of cardiovascular events. The aim of the present study was therefore to identify potential biomarkers in circulating cells reflecting atherosclerotic lesion progression in the vessel wall. We performed gene arrays on circulating leucocytes from atherosclerosis prone *Apoe*^−/−^ mice with increasing ages, using C57BL/6 mice as healthy controls. We identified fatty acid binding protein 4 (FABP4) mRNA to be augmented in mice with established disease compared with young *Apoe*^−/−^ or controls. Interestingly, the transcript FABP4 correlated significantly with lesion size, further supporting a disease associated increase. In addition, validation of our finding on protein level showed augmented FABP4 in circulating leucocytes whereas, importantly, no change could be observed in plasma. Immunofluorescence analysis demonstrated FABP4 to be present mainly in circulating neutrophils and to some extent in monocytes. Moreover, FABP4-positive neutrophils and macrophages could be identified in the subintimal space in the plaque. Using human circulating leucocytes, we confirmed the presence of FABP4 protein in neutrophils and monocytes. In conclusion, we have showed that cellular levels of FABP4 in circulating leucocytes associate with lesion development in the experimental *Apoe*^−/−^ model. The increased expression is primarily localized to neutrophils, but also in monocytes. We have identified FABP4 in leucocytes as a potential and easy accessible biomarker of atherosclerosis which could be of future clinical relevance.

## Introduction

Atherosclerosis is a systemic disease of the arterial system characterized by lipid accumulation and inflammation in the vessel wall [1–3]. The disease is silent during decades until a usually sudden acute clinical manifestation occurs, such as a myocardial infarction or stroke. Although risk scoring models such as the Framingham model offers some individual prediction, many adverse cardiovascular events do occur in undiagnosed and asymptomatic individuals. Several inflammatory mediators in the circulation associate with increased cardiovascular risk and have been proposed as biomarkers, even though they only rarely add to the Framingham risk score (FRS). The utility of CRP is under debate [4, 5] and several other risk markers, including adiponectin, MMP-9 and IL-18, are being investigated [6]. It is important to note that FRS classifies risk over a period of 10 years, rather than in the near future within 1 year. A model that accurately calculates near-term risk of adverse cardiovascular events in diverse populations is still lacking [7]. This has prompted a search for novel biomarkers of atherosclerotic disease to improve identification of high risk patients by adding information about short-term risk to current risk prediction models.

We suggested that circulating inflammatory cells may carry signals with biomarker potential that may reveal progression of atherosclerotic disease, which is an important underlying risk factor for developing adverse cardiovascular events in the near future. We therefore investigated potential biomarkers reflecting disease development in circulating leucocytes using the atherosclerosis prone *Apoe*^−/−^ mouse model. To cover different stages of plaque development, from onset of disease with fatty streak formation to presence of late stage atherosclerotic lesions, the atherosclerotic mice were investigated at three different time-points. From an unbiased strategy, we identified cellular FABP4 expression in circulating leucocytes to be associated with the extent of atherosclerosis.

## Materials and methods

### Animals

All animal experiments were approved by the regional ethics committee for animal research in Stockholm [N82/09] and conform to the Directive 2010/63/EU of the European Parliament. Four sets of female *Apoe*^−/−^ and C57BL/6 mice were used. All mice were fed standard chow (R70, Lantmännen, Stockholm, Sweden).

### Lesion analysis

Three groups of *Apoe*^−/−^ mice, killed at 12, 20 and 32 weeks, and one group of C57BL/6 control animals killed at 85 weeks of age were studied. All animals were killed under CO_2_, blood was collected by cardiac puncture and sampled in EDTA tubes. The vasculature was perfused with sterile PBS before the descending thoracic aorta was dissected. The aortas were fixed in 4% formaldehyde, opened longitudinally, pinned onto wax plates, and stained with Sudan IV (Merck AG, Darmstadt, Germany). Aortic lesion areas were calculated with Leica Q500MC software.

### Plasma analysis

Alanine aminotransferase (ALT), creatine kinase myocardial band (CKMB) and creatinine (CRSC) were determined by Vitros DT60II (Ortho-Clinical Diagnostics, Raritan, NJ, USA).

### RNA, microarrays and RT-PCR

RNA from blood leucocytes was isolated using RNeasy-kit according to the manufacturer's protocol (QIAGEN Inc., Hilden, Germany) with a DNase step. RNA quantity and quality were assessed using a Bioanalyzer (Agilent Technologies, Palo Alto, CA, USA). Equal amount of RNA from individual mice was pooled (*n* = 6–8 per group) in each age group. Each group was analysed by Affymetrix microarray, MOE430A. In addition, specific transcripts were analysed by TaqMan RT-PCR with ABI Prism7700 sequence detector and software (Applied Biosystems, Foster City, CA, USA) in the individual RNA samples to validate array findings. An additional group was added to investigate FABP4 mRNA in *Apoe*^−/−^ and C57Bl6 aged 8 weeks. The primer/probe set used were; FABP4: Mm00445878_m; CD36: Mm00445878_m1 and as house-keeping gene, HPRT: Mm00446968_m1. Data were analysed on the basis of the relative expression with the formula 2^−ΔΔCT^, where ΔΔCT = ΔCT (sample)−ΔCT (calibrator=average CT values of all sample values within each group), and ΔCT is the CT value of the house-keeping gene subtracted from the CT value from the sample.

### Differential leucocyte count and ELISA assay of FABP4

Blood was drawn in EDTA tubes from an additional set of *Apoe*^−/−^ mice aged 10–11 weeks, 20–22 weeks and 35–37 weeks (*n* = 7–8/group). To analyse total numbers of WBC and platelets, differential counts of lymphocytes, monocytes, granulocytes and platelets were run on an Animal Blood Counter machine (ScandiVet, Enköping, Sweden). Plasma was collected after 1500 × *g* centrifugation for 10 min., RBC lysed with EL-buffer (Qiagen, Sollentuna, Sweden) and protein from the leucocytes isolated with T-Per Tissue Protein Extraction Reagent with recommended protease inhibitors (Thermo Scientific, Rockford, IL USA). Plasma levels and cellular levels of FABP4 protein were determined by mouse FABP4 ELISA, MBS366055 (MyBioSource, San Diego, CA, USA).

### Flow cytometry

*Apoe*^−/−^ and C57BL/6 mice, 12, 20 and 32 weeks of age (*n* = 8 per group) were killed and blood collected for FACS analysis. After lysis of red blood cells, Fc-receptor blockage was performed with anti-CD16/CD32 (BD Pharmingen, San Diego, CA, USA), before staining with primary conjugated antibodies to investigate the monocyte populations (see list below). Data were acquired, immediately after staining, using a CyAn ADP cytometer (Beckman Coulter, High Wycombe, UK) and analysed with the software Summit (DAKO, CO, USA). The following antibodies were used; antimouse CD45R/B220-PE (Biolegend, Uithoorn, The Netherlands), Ly6G-Pacific Blue, CD11b PE-Cy7, CD11c-APC, Ly6C-FITC, CD49b(DX5)-PE, NK1.1-PE, CD3-PE (BD, San Jose, CA, USA).

### Immunofluorescence staining

Leucocytes were isolated and centrifuged at 400 r.p.m., 5 min., onto Super-Frost glass slides (100,000 cells/spot) before fixation in ice-cold paraformaldehyde (PFA) for 10 min. Cells were blocked with avidin/biotin and 5% serum, following incubation with primary antibody against FABP4 in 0.1% tween at 4°C over night. The following day, secondary antibody anti-rabbit DyLight488 (1:200) was added for 1 hr. For double staining, a second primary antibody (see list and vendors below) was added for 1 hr in RT, followed by a biotinylated antibody before streptavidin labelled DyLight594 (1:200). Nuclei were stained with DAPI (Sigma-Aldrich, Saint-Louis, MO, USA) before mounting and analysis with a Leica TCS-SP5 confocal microscope.

In addition, 10-μm cryostat sections cut from the aortic root were analysed for the presence of FABP4 in combination with Ly6G or CD68, using the same staining protocol as above. For all stainings, appropriate isotype controls were used. Antibodies used: *Primary*: FABP4 rabbit antimouse, CD3 hamster antimouse (Abcam, Cambridge, MA, USA), B220 rat antimouse, Ly6G rat antimouse (Biolegend, Uithoorn, The Netherlands) and CD68 rat antimouse (AbD Serotec, Oxford, UK). *Secondary*: Biotinylated horse anti-rat, biotinylated goat anti-hamster, streptavidin-DyLight 594 and goat anti-rabbit Alexa488 (Vector Labs, Peterborough, UK).

### Immunofluorescence staining of human leucocytes

Blood was drawn from four healthy controls in EDTA tubes as approved by Stockholm Regional Ethical committee [D.nr. 2005/880-31/3]. Leucocytes were isolated and centrifuged at 400 r.p.m., 5 min., onto Super-Frost glass slides (100,000 cells/spot) before fixation in PFA for 10 min. Following the protocol above, staining was made with the following antibodies: Primary: FABP4 rabbit anti-human (Abcam), CD66b mouse anti-human (Fitzgerald, Acton, MA, USA) and CD14 mouse anti-human (Becton Dickinson, Franklin Lakes, NJ, USA). Secondary: biotinylated horse antimouse, streptavidin-DyLight594, and goat anti-rabbit Alexa488 (Vector Labs, Peterborough, UK).

## Results

### Increased levels of FABP4 mRNA in circulating leucocytes in Apoe^−/−^ mice with progression of atherosclerosis - independent of ageing

**Fig. 1:**
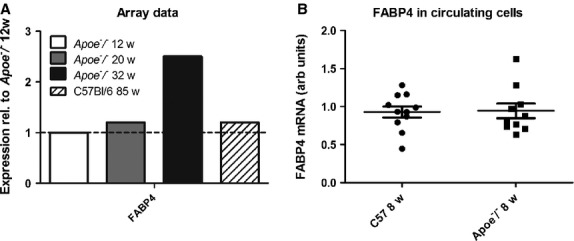
FABP4 mRNA levels in circulating leucocytes. (**A**) Gene arrays on circulating leucocytes show increased mRNA levels of FABP4 in 20 and 32 weeks old *Apoe*^−/−^, as compared to 12 weeks old *Apoe*^−/−^. C57BL/6, 85 weeks of age, were used as controls. One array per age group was run on pooled RNA (*n* = 6–8) from circulating leucocytes. (**B**) mRNA levels of FABP4 in circulating leucocytes from *Apoe*^−/−^ and C57Bl6 (8 weeks of age) measured by RT-PCR TaqMan (*n* = 10–11).

Gene expression profiles in leucocytes from atherosclerotic *Apoe*^−/−^ mice were analysed to identify if any atherosclerosis-related changes could be detected in circulating cells. The atherosclerotic mice were investigated at three different time-points, 12, 20 and 32 weeks of age, to cover different stages of plaque development. To adjust for any changes correlating to ageing rather than atherosclerosis, the non-atherosclerotic C57BL/6 mouse was used as control. When comparing the transcript profiles between *Apoe*^−/−^ and control mice, we revealed FABP4 to be the most changed transcript in the circulating leucocytes (Fig. 1A). The transcript level of FABP4 between young (8 weeks old) *Apoe*^−/−^ and control mice did not differ, as measured by real-time RT-PCR (Fig. 1B). Neither, ALT, CKMB or CRSC differed between the mice groups, indicating normal status of liver, heart and kidney (data not shown).

### FABP4 mRNA expression in circulating leucocytes correlates with lesion size

**Fig. 2:**
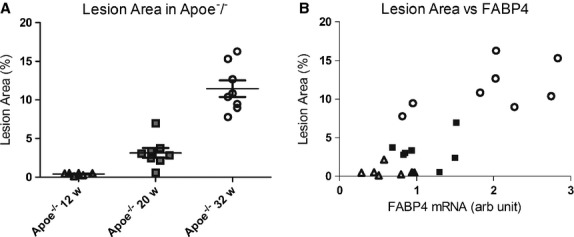
Levels of FABP4 mRNA in circulating leucocytes correlates with extent of atherosclerotic lesion in aorta. (**A**) Percent lesion area of total vessel area (*n* = 6–8 per group). (**B**) mRNA levels of FABP4 in circulating leucocytes from *Apoe*^−/−^ (12, 20 and 32 weeks of age) measured by RT-PCR TaqMan and correlated with percentage of lesion size (*r* = 0.800, ***P < 0.0001; *n* = 22).

To validate our array finding, real-time RT-PCR was performed from isolated leucocyte RNA of the individual *Apoe*^−/−^ mice. For quantitative lesion measurement, dissected thoracic aortas were pinned and stained with Sudan IV to measure lesion percentage. The lesion percentage was as expected with a mean of 0.4% in the 12 weeks age group, 3% in the 20 week and 11% in the 32 weeks age group (Fig. 2A). Correlation of lesion size in aorta and mRNA expression of FABP4 in circulating leucocytes gave an *r* = 0.800, ***P < 0.0001 (Fig. 2B).

### FABP4 and its relation to CD36 on mRNA level

Our time-course study of circulating leucocytes also identified an increase in CD36 mRNA level, positively correlated with FABP4 mRNA level (*r* = 0.594 **P < 0.001) and to lesion size (*r* = 0.675, ***P < 0.0001; Supporting Information Figure S1A and B). However, the correlation with disease development was less robust than that observed with FABP4.

### The FABP4 protein is augmented in circulating leucocytes and unchanged in plasma in Apoe^−/−^ mice

**Fig. 3:**
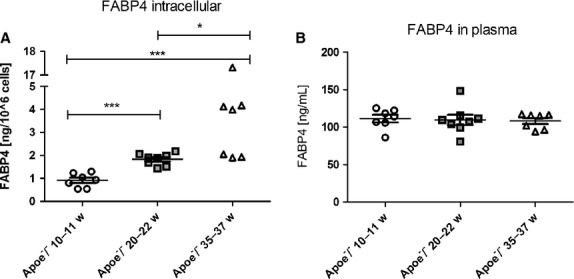
FABP4 protein level increases in circulating leucocytes in *Apoe*^−/−^ with disease progression, but is stable in plasma. (**A**) Cellular protein level in circulating leucocytes of FABP4 in *Apoe*^−/−^ (10–11, 20–22 and 35–37 weeks of age) as measured by ELISA (*n* = 7–8 per group). (**B**) FABP4 concentrations in plasma in *Apoe*^−/−^ (10–11, 20–22 and 35–37 weeks of age) as measured by ELISA (*n* = 7–8 per group). ***P < 0.0001 *versus Apoe*^−/−^ 20w, *P < 0.05 *versus Apoe*^−/−^ 32w. ns indicates not significant.

We repeated our time-course study, this time for protein analysis and differential count. Blood was drawn in EDTA tubes from *Apoe*^−/−^ mice (10–11, 20–22 and 35–37 weeks of age), plasma collected and protein extracts from leucocytes isolated to analyse leucocyte FABP4 protein content by ELISA. One aliquot of the sample was analysed in parallel by differential count of leucocytes. ELISA analysis demonstrated a cellular increase in FABP4, with no corresponding change in protein levels detected in the plasma comparing the different groups (Fig. 3A and B). The plasma concentrations of FABP4 in C57BL/6 were not altered with ageing, comparing 10, 20 and 32 weeks of age (data not shown).

Differential leucocyte cell counts in *Apoe*^−/−^ did not reveal any major changes in total cell numbers, except for an increase in platelet concentration (Supporting Information Table S1). From these analyses, we concluded that the changes in FABP4 mRNA levels and protein expression were not due to increases of a specific leucocyte population.

### The inflammatory monocyte subset constitutes the major part of the monocytic population preceding advanced stages of atherosclerosis

Flow cytometric analyses were performed on whole blood. Monocytes were identified as CD3^−^, B220^−^, DX5^−^ NK1.1^−^ C11c^mi^, CD11b^+^, Ly6G^−^ and Ly6C^+^. The monocytes were further analysed as Ly6C^hi^ or Ly6C^lo^. In *Apoe*^−/−^ 12 weeks and 20 weeks of age, the inflammatory Ly6C^hi^ monocyte subtype constituted the greater part of all the CD11b^+^Ly6C^+^ monocytes. This distribution changed in the 32-week-old animals, where the percentage of Ly6C^hi^ and Ly6C^lo^ was equal (Supporting Information Figure S2A and C). In C57BL/6 mice, the percentage between the inflammatory and non-inflammatory monocyte populations did not differ significantly (Figure S2B).

### FABP4 is expressed by neutrophils and monocytes

**Fig. 4:**
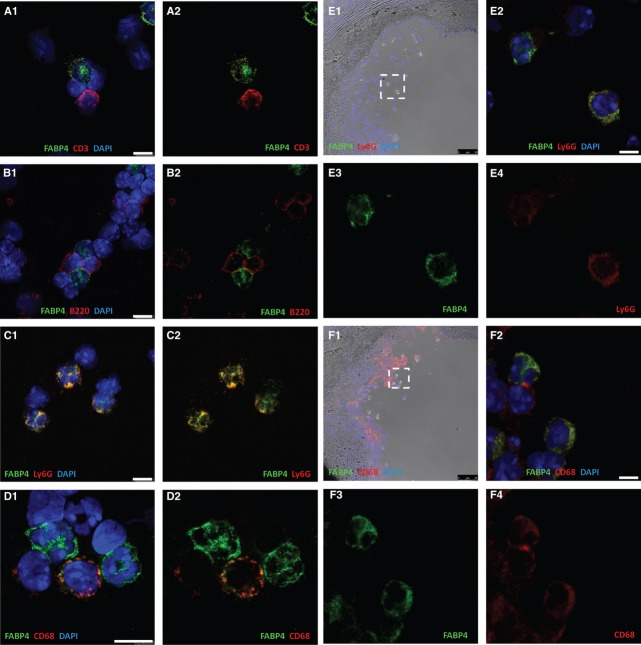
Immunofluorescent staining of FABP4 and leucocytes in the circulation of *Apoe*^−/−^ *mice*. (**A**–**D**) Immunofluorescent staining of leucocytes from *Apoe*^−/−^ 32 weeks of age with FABP4 (in green) with the T cell marker CD3 (**A**), the B cell marker B220 (**B**), the neutrophil marker Ly6G (**C**) and the monocyte/macrophage marker CD68 (**D**) (all in red). Nuclei stained blue. White bar is 10 μm. (**E**–**F**) Immunofluorescent staining of atherosclerotic lesions from *Apoe*^−/−^ 32 weeks of age with FABP4 (in green) in combination with the neutrophil marker Ly6G (**E**) and the monocyte/macrophage marker CD68 (**F**). Nuclei stained blue. White bar is 10 μm.

To determine cell type in the circulation with FABP4 expression, we performed immunohistochemical analysis. Leucocytes from both the *Apoe*^−/−^ and C57BL/6 were deposited by cytospin and stained with FABP4 antibody, combined with cell markers for T cells (CD3), B cells (B220), monocytes (CD68) and neutrophils (Ly6G). Neither T, nor B cells, showed any expression of FABP4 in either of the mice strains at the different ages analysed (12, 20 and 32 weeks of age; Fig. 4A and B). The neutrophils turned out to be the cell type in the circulation with strongest FABP4 expression in all ages analysed and in both strains (Fig. 4C). Monocytes, defined as CD68^+^ cells also stained with FABP4, but to a lesser extent (Fig. 4D).

In murine atherosclerotic lesions, FABP4 was found to be localized mainly to the subintimal space (Fig. 4E and F). Both Ly6G^+^ neutrophils and CD68^+^ macrophages positive for FABP4 were identified in the lesion (Fig. 4E and F).

### In humans, FABP4 is expressed by neutrophils and monocytes in the circulation

**Fig. 5:**
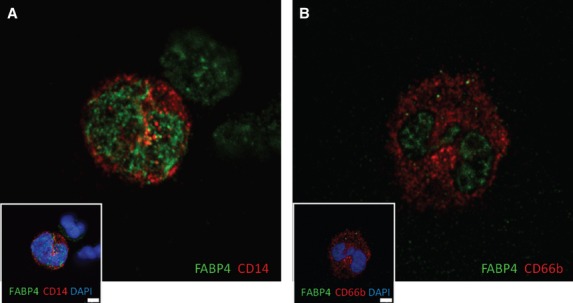
Human neutrophils and monocytes are positive for FABP4. Human leucocytes were stained with FABP4 (green) and the monocyte marker CD14 (**A**) or the neutrophil marker CD66b (**B**). Nuclei stained blue with DAPI, as seen in the small imbedded photos in the left hand corner of A and B. White bar 5 μm.

From blood samples drawn from healthy human beings, peripheral circulating blood cells were purified and analysed in the same way as with the murine cells. IHC analysis was performed with the neutrophil marker CD66b (CEACAM8) and the monocyte marker CD14, to investigate human expression pattern. Interestingly, both cell populations turned out to be positive for FABP4 (Fig. 5A and B).

## Discussion

The aim of our study was to identify markers in the circulation outside the plaque itself which correlate to the development of atherosclerotic disease before onset of symptoms. By using unbiased transcript profiling to map changes over time in circulating leucocytes in *Apoe*^−/−^ mice and non-atherosclerotic control mice, we identified FABP4 in circulating neutrophils and to a weaker extent in monocytes as the major changed transcript with regard to progression of atherosclerotic disease. We also demonstrated that increased cellular FABP4 protein is specifically upregulated in circulating leucocytes and that no spill-over to plasma occurred as levels of FABP4 in plasma remained unchanged. These findings suggest that cellular mRNA signatures may serve as early biomarkers. Moreover, we demonstrated FABP4 in neutrophils, as well as in macrophages in the murine atherosclerotic lesion. In addition, neutrophils and monocytes from healthy human blood donors are also shown to contain FABP4.

Several studies, both in experimental models and human data, have shown FABP4 to be involved in atherosclerotic lesion progression [8–11]. FABP4 is a 15 kD cytoplasmic protein binding hydrophobic ligands, such as unsaturated and saturated fatty acids and eicosanoids, but the exact biological function(s) are still unknown [12]. FABP4 was initially identified as a key protein in adipocyte differentiation and has later been shown in macrophages, foam cells [13] and immune competent dendritic cells [14].

We previously demonstrated augmented FABP4 expression in the human atherosclerotic lesion to be associated with unstable plaque phenotype [15], this was later confirmed by others [16, 17]. In addition, levels of FABP4 in established atherosclerotic plaques correlate with increased risk for cardiovascular events during follow-up, independent from medication, serological or general risk factors [16, 17]. Although it is of major interest to understand complex processes within the lesion to gain knowledge of the pathogenesis, the lesion *per se* is quite impractical for direct access to measure biomarkers. The fact that atherosclerosis is a chronic systemic inflammatory disease may imply that circulating blood cells possess the potential to mirror an ongoing atherosclerotic disease process. It would, in fact, be advantageous if specific biomarker measurements could be performed on circulating blood cells because they are easy to sample, and the subsequent mRNA/protein measurements are relatively quick and cheap. A recent study suggested the future use of a ‘multi-gene biomarker model’ across different sets of leucocytes when predicting early stage atherosclerosis in asymptomatic patients [18]. Our present study using gene transcript profiling and protein measurements in circulating blood cells from an experimental model, demonstrate FABP4 to be a robust marker of the extent of atherosclerosis. Interestingly, the protein level of FABP4 in the plasma of neither control nor *Apoe*^−/−^ mice change with age or with disease progression, suggesting that cellular upregulation in leucocytes is an early event in disease progression. The lack of an increase in plasma protein levels suggests that plasma FABP4 levels may not necessarily derive from circulating leucocytes or may be a later event downstream of cellular upregulation. Nevertheless, cellular measurement may be preferred for early detection. In addition, we also observed a correlation with the membrane glycoprotein CD36 mRNA in circulating cells to lesion size and to FABP4 expression. CD36 is a class B scavenger receptor known to be expressed on a wide variety of cells, in particular on monocytes and macrophages, and has a role in uptake of oxidatively modified low-density lipoprotein (LDL) as well as other ligands. The possible mechanistic role of the relationship between FABP4 and CD36 is not clarified from this study, but could speculatively involve increased cellular lipid uptake and thus increased intracellular lipid loading but further investigations are needed.

In the circulation, we found FABP4 to be present mainly in neutrophils but also in monocytes. Neutrophils communicate avidly with other immune cells such as monocytes/macrophages, T lymphocytes and dendritic cells but also endothelial cells and smooth muscle cells, *via* direct cell contact and the release of mediators. In addition, neutrophils are producers of the potent leucocyte chemoattractant lipid mediator LTB4 [19] and FABP4 has the capacity to bind inflammatory lipid precursors, leukotrienes and stabilize LTA4 *in vitro* [20]. We have previously shown a correlation in the human atherosclerotic lesion between FABP4 mRNA and the leukotriene enzymes 5-LO and LTA4H mRNA [15]. It is therefore tempting to speculate that the increased expression of FABP4 within these cells, would relate to increased production of LTB4. In the murine atherosclerotic plaque, we observed FABP4-positive neutrophils in the shoulder regions and close to the subintimal space in the murine plaque. Given their short life span in inflamed tissues, neutrophils have retained little attention in atherosclerosis up until recently [21, 22]. New evidence suggests that these cells can contribute and play a major role in orchestrating the inflammatory response in atherosclerotic disease. By using intra-vital microscopy it has been shown that a majority of leucocytes interacting with the endothelium on lesion shoulders are neutrophils, which would imply recruitment of these cells to the plaque [23, 24]. Once in the vessel wall, neutrophils are known to rapidly become apoptotic [25] and release ‘find-me’ and ‘eat-me’ signals to attract macrophages for scavenging [26]. In addition to FABP4-positive neutrophils in the lesion, we also found FABP4-positive macrophages throughout the lesion. Compared with the CD68-positive cells in the circulation, the macrophages within the lesion express more FABP4. We speculate that, in addition to the known upregulation of FABP4 during foam cell formation, scavenging of apoptotic cells might result in augmented FABP4 expression in the lesional macrophages/foam cells.

As mentioned previously, immunohistochemistry of the circulating leucocytes from control and *Apoe*^−/−^ mice reveal a weak FABP4 staining in some monocytes. The monocytes can be divided into the more inflammatory Ly6C^high^ and the less inflammatory Ly6C^low^ subpopulation [27]. By flow cytometry, we observed a significant higher percentage of the Ly6C^high^ subset in 12 and 20 weeks old *Apoe*^−/−^ mice. This is in contrast to later stage of disease and during all time-points investigated for C57/BL6, where the distribution of Ly6C^high^ and Ly6C^low^ is similar. Both subsets are known to infiltrate the atherosclerotic lesions, although Ly6C^high^ are more robustly recruited [28]. In the future, it would be of interest to pinpoint the activation status of the different FABP4-positive monocyte/macrophage and neutrophil populations in the circulation as well as within the murine lesion.

In human circulating leucocytes, we also detected FABP4 protein in monocytes and neutrophils. Studies have demonstrated neutrophils at the site of plaque erosion or rupture, in specimens from patients with unstable angina and in samples from patients having an acute MI [29, 30]. Furthermore, high neutrophil numbers in carotid atherosclerotic plaques have been associated with unstable plaques [29]. These findings make it highly relevant to further investigate the role of neutrophils containing FABP4 and their possible role as disease biomarker in atherosclerosis.

In conclusion, our main findings in this study show (a) cellular levels of FABP4 in the circulating leucocytes to associate with lesion development in *Apoe*^−/−^ and (b) FABP4 to be expressed by the neutrophils and monocytes in the circulation. On the basis of these findings, we demonstrate the utility of cellular RNA and protein levels as a biomarker of atherosclerosis in this experimental *Apoe*^−/−^ model. In addition, we show a similar expression pattern in human circulating cells. Further investigations in humans will have to be performed to determine a possible clinical utility of our findings.

## Supporting information

**Figure S1** Levels of CD36 mRNA in Circulating Leucocytes Associates with FABP4 mRNA and Extent of Atherosclerotic Lesion in Aorta. (**A**) Correlation of mRNA levels of FABP4 and CD36 in circulating leucocytes from *Apoe*^−/−^ (12, 20 and 32 weeks of age) as measured by RT-PCR TaqMan, n = 22 (r = 0.594 ***P* < 0.001). (**B**) Correlation between lesion area and CD36 mRNA, n = 22 (r = 0.675, ****P* < 0.0001).

**Figure S2** Monocyte Subsets in *Apoe*^−/−^ and C57BL/6. Flow cytometry on circulating leucocytes from *Apoe*^−/−^ (**A**) and C57BL/6 (**B**) (12, 20 and 32 weeks of age). Graphs showing the percentage of the inflammatory monocyte subtype Ly6C^High^ *versus* Ly6C^low^. The monocytes were defined as CD3^−,^ B220^−^, DX5^−^ NK1.1^−^ C11c^low^, CD11b^+^ and Ly6G^−^ and further divided into Ly6C^High^ or Ly6C^low^. (**C**) Representative flow cytometry plots showing CD11b^+^ Ly6C^High^ and CD11b^+^ Ly6C^low^ from *Apoe*^−/−^ 12 and 32 weeks of age (***P <* 0.001, ns indicates not significant).

**Table S1:** Differential Blood Count in *Apoe*^−/−^ and *C57BL/6*.
