# Corrigendum

**DOI:** 10.1111/jcmm.12083

**Published:** 2013-06-20

In 1, authors Gabrielle Paulsson-Berne and Anders Gabrielsen contributed equally to the study.

The authors wish to apologise for any misunderstanding or inconvenience caused.
