# Supplementary figures and images for: Fatty acid binding protein 4 in circulating leucocytes reflects atherosclerotic lesion progression in Apoe−/− mice
Source: J Cell Mol Med. 2013 Feb 7;17(2):303–10. doi: 10.1111/jcmm.12011 (PMC3822593; doi:10.1111/jcmm.12011)

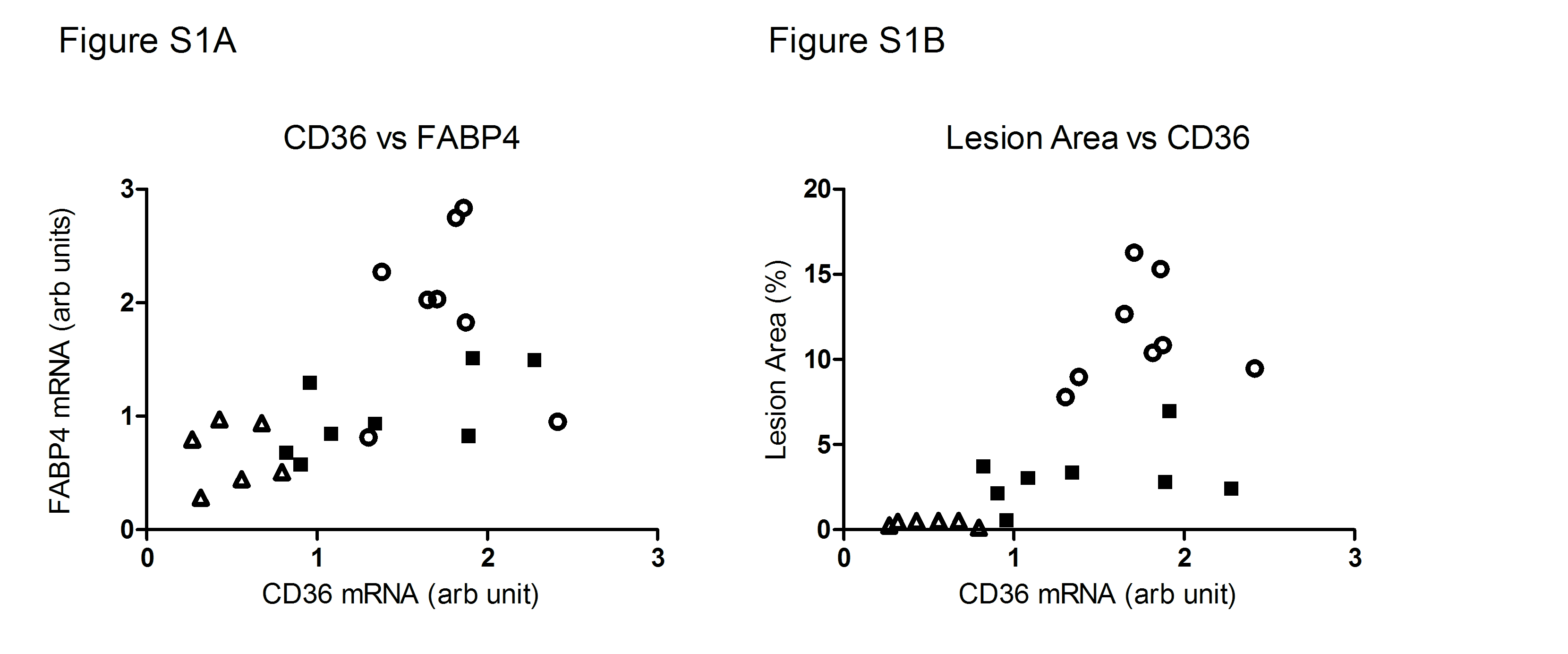

Supplement: Supplementary file 1 [file jcmm0017-0303-SD1.tif]

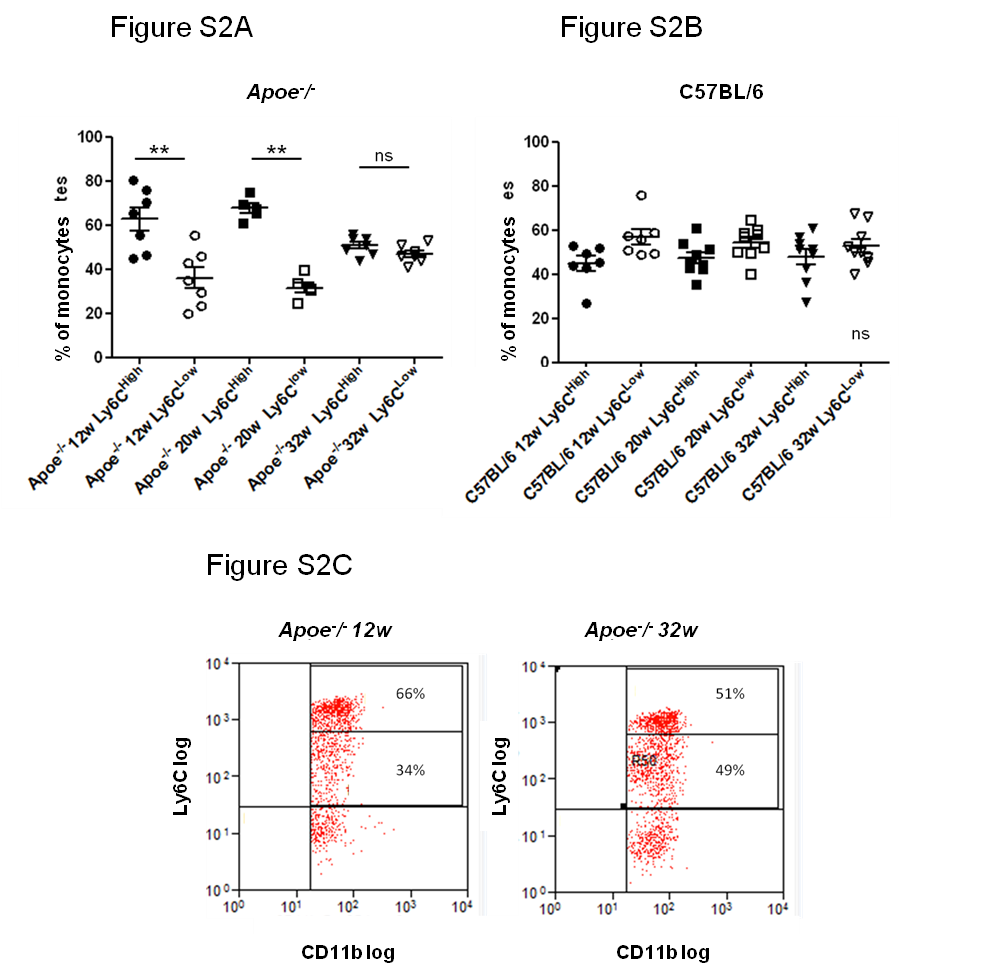

Supplement: Supplementary file 2 [file jcmm0017-0303-SD2.tif]
