# Supplementary material for: Fatty acid binding protein 4 in circulating leucocytes reflects atherosclerotic lesion progression in Apoe−/− mice
Source: J Cell Mol Med. 2013 Feb 7;17(2):303–10. doi: 10.1111/jcmm.12011 (PMC3822593; doi:10.1111/jcmm.12011)
Supplement: Supplementary file 3 [file jcmm0017-0303-SD3.docx]

**Supplemental Table I. Differential Blood Count in** ***Apoe^-^/^-^ and C57BL/6***

|  |  |  | | | ***Apoe^-^/^-^*** | |  | |  | |  | ***C57BL/6*** |  |
| --- | --- | --- | --- | --- | --- | --- | --- | --- | --- | --- | --- | --- | --- |
| **Cell Types**  [10^9^/L± stddev] | | |  | **10-11w** | | **20-22w** | | **35-37w** | |  | **10-11w** | **20-22w** | **35-37w** |
| ***WBC*** | | |  | 11±2,5 | | 8,1±2,1* | | 8,7±3,3 | |  | 8,1±2,8 | 6,5±1,1 | 7,8±2,0 |
| ***Lymphocytes*** | | |  | 7,5±1,8 | | 5,9±1,5 | | 6,0±2,4 | |  | 6,1±1,6 | 5,0±0,7 | 5,8±1,4 |
| ***Monocytes*** | | |  | 0,6±0,2 | | 0,4±0,2 | | 0,4±0,2 | |  | 0,4±0,1 | 0,3±0,1 | 0,3±0,1 |
| ***Granulocytes*** | | |  | 2,0±0,7 | | 2,0±0,7 | | 2,2±0,8 | |  | 1,8±1,2 | 1,2±0,5 | 1,7±0,7 |
| ***Platelets*** | | |  | 780±200 | | 1200±140****** | | 1500±400****** | |  | 1100±240 | 1200±160 | 1200±210 |

WBC: white blood cells
